# Association between keratoconus disease severity and repeatability in measurements of parameters for the assessment of progressive disease

**DOI:** 10.1371/journal.pone.0228992

**Published:** 2020-02-14

**Authors:** Ingemar Gustafsson, Anders Bergström, Anna Cardiakides Myers, Anders Ivarsen, Jesper Hjortdal

**Affiliations:** 1 Department of Clinical Sciences Lund, Ophthalmology, Lund University, Skane University Hospital, Lund, Sweden; 2 Department of Clinical Sciences, Faculty of Medicine, Lund University, Lund, Sweden; 3 Department of Ophthalmology, Aarhus University Hospital, Aarhus, Denmark; Wenzhou Medical University Eye Hospital, CHINA

## Abstract

**Background:**

Progressive keratoconus can lead to severely impaired vision, but there is currently no consensus on the definition of progressive disease. Errors in the measurement of the parameters commonly used to establish progressive disease were evaluated in an attempt to determine the limits at which a true change in the values can be detected. The possible association between measurement error and disease severity was also investigated to evaluate the need for limits based on disease severity.

**Methods:**

Sixty-one eyes were studied in 61 patients with keratoconus. Four replicate measurements were made in each patient using a Scheimpflug-based tomographic system (denoted the PC) and an auto-keratometer (denoted the AK). The repeatability coefficient, i.e., the level below which differences between two measurements are found in 95% of paired observations, was calculated. Patients were further divided into three groups based on disease severity (parameter magnitude).

**Results:**

Increasing magnitude of all the keratometric parameters investigated was significantly associated with increasing measurement errors, and thus worse repeatability. The maximum keratometry value (Kmax) was the least repeatable parameter (1.23 D, 95% CI 1.11–1.35 D) and showed the strongest association between parameter magnitude and measurement error. The repeatability coefficient ranged between 0.32 and 1.62 D, depending on disease severity. The most repeatable parameter was the flattest central keratometry value (K1), measured with the PC (0.51 D, 95% CI 0.46–0.56 D) and the AK (0.54 D, 95% CI 0.48–0.59 D). K1 showed the weakest association between parameter magnitude and measurement error. The repeatability coefficient for K1 ranged between 0.40 and 0.54 D when using the PC, and between 0.34 and 0.70 D when using the AK in the three groups.

**Conclusions:**

The association between the magnitude of the keratometric parameters and their measurement errors suggests that limits should be based on disease severity to ensure reliable detection of progressive keratoconus. Further studies are, however, required.

## Introduction

Keratoconus is a corneal disease that can lead to severely impaired vision. It usually manifests in adolescents, and can have a significant negative impact on the quality of life [1]. In 2003 corneal crosslinking (CXL) emerged as a novel treatment for stabilizing progressive keratoconus [2]. Today, there is growing evidence that the progression of keratoconus can be halted by CXL [3] [4] [5], preventing further visual deterioration, and reducing the need for penetrating keratoplasty [6] [7]. Recent approval of CXL for the treatment of progressive keratoconus by the US FDA has confirmed the importance of this treatment.

It is important to detect keratoconus early, and to monitor it carefully for any signs of progression, so that CXL can be performed when appropriate. The instruments currently available for corneal imaging allow early detection of keratoconus [8], but there is no consensus on the definition of progressive keratoconus, which is the common indication for CXL. In 2015, it was reported that a consistent steepening of anterior or posterior corneal curvature and corneal thinning were suggestive of progressive disease [9]. It was also stated that the magnitude of the change should be greater than the measurement error. However, no specific limits were suggested for the magnitude of the change. Differences in measurements can result from a true change, or measurement error due to the intrinsic accuracy of the instrument. In order to define the level at which a true change can be suspected based on measurements, the repeatability coefficient (R) must be calculated. However, studies on the repeatability of measurements of topographic and tomographic parameters in keratoconus are often inconsistent. Some reports have suggested poorer repeatability in cohorts with more advanced disease [10] [11]. An increasing measurement error with increasing disease severity could explain some of the differences reported in repeatability, necessitating appropriate methods of calculating repeatability [12].

To the best of our knowledge, no studies have been performed to investigate the possible relation between measurement errors and the magnitude of parameters reflecting disease severity. The aim of this study was therefore to investigate this relation, and to calculate repeatability limits, based on disease severity, that could indicate a true change between measurements. Measurements of the anterior and posterior corneal curvature and corneal thickness were made using a Scheimpflug-based device, which is probably the most commonly used instrument in the management of keratoconus. Measurements of the anterior corneal curvature using auto-keratometry were also evaluated. As far as we know, auto-keratometry has not previously been evaluated in the management of keratoconus, but its repeatability is high in healthy corneas [13].

## Subjects and methods

This study was conducted at the Department of Ophthalmology, Skåne University Hospital, Lund, Sweden, according to the Declaration of Helsinki. The Regional Ethics Committee in Lund approved the study (No. 2015/373).

Patients with keratoconus fulfilling the inclusion criteria were enrolled in the study after signing an informed consent form. The inclusion criteria were: keratoconus with no history of other ocular pathology or prior ocular surgery, and age > 18 years. Contact lens wear was discontinued at least 2 weeks before the measurements were made. Patients with corneal scarring were excluded. Pregnant and breastfeeding women were also excluded.

Keratoconus was diagnosed clinically, and by examination using a Scheimpflug-based device (see below). The sagittal curvature pattern, posterior and anterior elevation maps and corneal thickness pattern were assessed, in addition to information from the Belin-Ambrosio Enhanced Ectasia Display [14].

Sixty-one eyes in 61 patients were included. Only one eye was eligible for inclusion in 23 patients, due to prior CXL, penetrating keratoplasty or the presence of corneal scarring in the other eye. Computerized randomization was performed in the remaining 37 patients to select one eye for inclusion in the study (29 right eyes and 32 left eyes). Fifty-four participants were male, and 7 female, and the mean age was 29 years (range 18–49 years).

Four replicate measurements were made by the same examiner (IG) using the Pentacam HR system (Pentacam HR, version 1.20r10, Oculus Optikgeräte GmbH, Wetzlar, Germany). Patients were instructed to blink but not to lean back between measurements. Four replicate measurements were then made using auto-keratometry (NIDEK ARK-560A, NIDEK Co. Ltd., Japan) under the same conditions and by the same examiner, using auto-alignment mode. Only examinations deemed “OK” by the Pentacam HR system and error-free by the NIDEK ARK-560A instrument were accepted.

### Instruments and parameters measured

The Pentacam HR (denoted PC) is a Scheimpflug-based tomographic system, the technical features of which have been described elsewhere [15]. The default setting of 25 images/s was used. The flattest central keratometry value (K1), the steepest central keratometry value (K2), the maximum keratometry value (Kmax), the posterior minimum radius (r-min) and the minimum corneal thickness (MCT) were measured with this instrument. K1 and K2 were measured in the central 3 mm zone.

The NIDEK ARK-560A (denoted AK) is a combined refractometer and keratometer. It captures a mire ring on the cornea on which analysis is based. K1 and K2 were obtained in auto-alignment mode using the standard 3.3 mm diameter zone of measurement.

### Statistical methods and calculations

IBM SPSS Statistics 22 for Windows (IBM Corporation, Armonk, NY, USA) and SAS Enterprise Guide 6.1 for Windows (SAS Institute Inc., Cary, NC, USA) were used for statistical analyses. Statistical significance was defined as a p-value of ≤ 0.05. Descriptive statistics are given as subject mean, standard deviation, median, and minimum and maximum values. Repeatability was assessed by calculating the within-subject standard deviation with 95% confidence intervals, the repeatability coefficient with 95% confidence intervals, intraclass correlation and the coefficient of variation [16] [17] [18]. Kendall’s Tau-b was used to analyse correlations between the mean and standard deviation of replicate measurements. Transformed (natural logarithm) data were analysed where appropriate. K1, K2 and Kmax values were divided into three groups based on parameter magnitude to give groups of as equal size as possible. Differences between coefficients of variation were assessed using a regression test [19]. Bland-Altman plots were used to analyse the agreement between the two instruments. Limits of agreement were calculated using a linear mixed model for replicate measurements [16]. A professional medical statistician was consulted.

### Definitions

- Repeatability: the variation in repeated measurements made on the same subject under identical conditions. The underlying values are assumed to be constant during the measurements [20].
- Within-subject standard deviation (S_w_): the square root of the mean of subject variance [17].
- Repeatability coefficient (R) (2.77 x S_w_): the difference between two measurements should be below this limit for 95% of pairs of observations [17].
- Coefficient of variation (CV): S_w_ divided by the overall mean [12][17].
- Intraclass correlation coefficient (ICC): (the variance between subjects) divided by (the variance between subjects plus the variance within a subject) [18].

## Results

### Repeatability of measurements

The value of ICC was high for all parameters, and the variability was attributed to differences between subjects, rather than within subjects. The CV was used for intra-instrument comparison of parameters. K1 showed the best repeatability (PC, CV = 0.41%) (AK = 0.43%), followed by K2 (PC, CV = 0.57%) (AK, CV = 0.50%), Kmax (CV = 0.80%), MCT (CV = 1.05%) and r-min (CV = 1.28%) (Table 1). In order to evaluate differences in repeatability between the instruments for K1 and K2 a regression test was performed to compare the CV for each parameter. No statistically significant differences were found in measurements between the PC and the AK instruments (K1, p = 0.130, K2, p = 0.498) (Table 2). The repeatability of K1 was then compared to that for K2 with both instruments, revealing a statistically significant higher repeatability for K1 than for K2 (PC, p = 0.002, AK, p<0.001) (Table 2). In parameter-specific units, the repeatability was best for K1 (PC, R = 0.51 D, 95% CI 0.46–0.56 D) (AK, R = 0.54 D, 95% CI 0.48–0.59 D), followed by K2 (PC, R = 0.76 D, 95% CI 0.68–0.83 D) (AK, R = 0.69 D, 95% CI 0.62–0.76 D), Kmax (R = 1.23 D, 95% CI 1.10–1.35 D), MCT (R = 14.2 μm, 95% CI 12.7–15.6 μm) and r-min (R = 0.18 mm, 95% CI 0.16–0.19 mm).

**Table 1:** Repeatability of the PC and AK measurements.

		n	Mean (SD)^a^	Median (Min–Max)^a^	S_w_ (95% CI)	Repeatability (95% CI)	ICC	CV (%)	Kendall’s Tau^b^	p^b^
**K1 (D)**										
PC		61	44.0 (3.13)	43.5 (38.9–60.2)	0.18 (0.16–0.20)	0.51 (0.46–0.56)	0.997	0.41^c^	0.239	0.008
	<43.0	20	41.4 (1.32)	41.4 (38.9–42.9)	0.14 (0.12–0.17)	0.40 (0.33–0.47)	0.988	0.35	0.092	0.597
	≥43.0 <44.5	20	43.6 (0.38)	43.4 (43.0–44.1)	0.21 (0.17–0.24)	0.57 (0.47–0.68)	0.753	0.47^c^	0.330	0.049
	≥44.5	21	46.8 (3.49)	45.3 (44.5–60.2)	0.19 (0.16–0.23)	0.54 (0.44–0.63)	0.997	0.41	0.302	0.057
AK		61	44.8 (3.09)	44.2 (40.7–62.2)	0.19 (0.17–0.21)	0.54 (0.48–0.59)	0.996	0.43^c^	0.230	0.016
	<43.5	18	42.3 (0.72)	42.2 (40.7–43.3)	0.12 (0.10–0.15)	0.34 (0.28–0.40)	0.971	0.29	-0.145	0.444
	≥43.5 <45.2	20	44.1 (0.42)	44.1 (43.5–44.7)	0.16 (0.14–0.19)	0.46 (0.37–0.54)	0.862	0.37	0.111	0.546
	≥45.2	23	47.4 (3.55)	46.7 (45.2–62.2)	0.25 (0.21–0.29)	0.70 (0.58–0.82)	0.995	0.53	0.051	0.746
**K2 (D)**										
PC		61	47.0 (4.23)	46.1 (41.4–67.8)	0.27 (0.25–0.30)	0.76 (0.68–0.83)	0.996	0.57^c^	0.305	0.001
	<44.8	20	43.3 (1.04)	43.6 (41.4–44.8)	0.13 (0.10–0.15)	0.35 (0.29–0.42)	0.985	0.29	-0.154	0.373
	≥44.8 <47.8	20	46.3 (0.94)	46.1 (44.8–47.6)	0.21 (0.17–0.24)	0.57 (0.47–0.67)	0.954	0.44^c^	0.338	0.040
	≥47.8	21	51.3 (4.32)	50.2 (47.8–67.8)	0.40 (0.33–0.47)	1.11 (0.92–1.31)	0.991	0.78	0.129	0.415
AK		61	47.6 (4.55)	46.6 (41.9–73.6)	0.25 (0.22–0.27)	0.69 (0.62–0.76)	0.997	0.50^c^	0.320	0.001
	<45.3	20	44.0 (0.96)	44.4 (41.9–45.2)	0.12 (0.095–0.14)	0.32 (0.26–0.38)	0.986	0.26	0.150	0.405
	≥45.3 <48.6	20	46.9 (0.98)	46.6 (45.3–48.3)	0.23 (0.19–0.27)	0.63 (0.52–0.74)	0.949	0.49	-0.091	0.608
	≥48.6	21	51.7 (5.38)	50.1 (48.6–73.6)	0.34 (0.28–0.40)	0.95 (0.78–1.12)	0.996	0.66	0.035	0.830
**Kmax (D)**										
PC		61	52.0 (6.14)	50.6 (42.5–77.1)	0.44 (0.40–0.49)	1.23 (1.10–1.35)	0.995	0.80^c^	0.532	<0.001
	<48.2	20	46.2 (1.62)	46.6 (42.5–48.0)	0.11 (0.094–0.13)	0.32 (0.26–0.37)	0.995	0.25	0.281	0.090
	≥48.2 <53.9	21	51.1 (2.03)	50.6 (48.2–53.9)	0.48 (0.40–0.56)	1.33 (1.10–1.56)	0.946	0.94	0.298	0.061
	≥53.9	20	58.8 (5.27)	57.3 (53.9–77.1)	0.59 (0.48–0.69)	1.62 (1.33–1.91)	0.988	1.00	0.316	0.052
**MCT (μm)**										
PC		61	485.5 (40.6)	483.0 (394.5–578.0)	5.11 (4.59–5.63)	14.2 (12.7–15.6)	0.984	1.05	0.134	0.129
**r-min (mm)**										
PC		61	4.93 (0.71)	4.93 (2.99–6.29)	0.063 (0.057–0.070)	0.18 (0.16–0.19)	0.992	1.28	-0.153	0.083

^a^ Subject Mean,

^b^ Subject SD versus subject Mean,

^c^ Calculated from transformed data, K1 (flattest central keratometry value), K2 (steepest central keratometry value), Kmax (maximum keratometry value), MCT (minimum corneal thickness), r-min (minimum posterior corneal radius), PC (Pentacam HR), AK (Nidek ARK 560A)

**Table 2:** Results of the regression test of CV for K1 and K2 within and between instruments.

	PC	AK	
K1 (D)	0.41	0.43	p = 0.130
K2 (D)	0.57	0.50	p = 0.498
	p = 0.002	p<0.001	

K1 (flattest central keratometry value), K2 (steepest central keratometry value)

PC (Pentacam HR), AK (Nidek ARK 560A)

### Stratified repeatability of measurements

The positive correlation between the magnitude of the measured parameter and its standard deviation corresponds to worsening repeatability of the measurements with increasing parameter magnitude (Table 1). Kmax showed the strongest such association (Kendall’s Tau-b = 0.532, p<0.001) followed by K2 (PC)(Kendall’s Tau-b = 0.305, p = 0.001)/(AK)(Kendall’s Tau-b = 0.320, p = 0.001) and K1 (PC)(Kendall’s Tau-b = 0.239, p<0.008)/(AK)(Kendall’s Tau-b = 0.230, p = 0.016). No significant association was found for MCT (Kendall’s Tau-b = 0.134, p = 0.129) or r-min (Kendall’s Tau-b = -0.153, p = 0.083).

**Fig 1:**
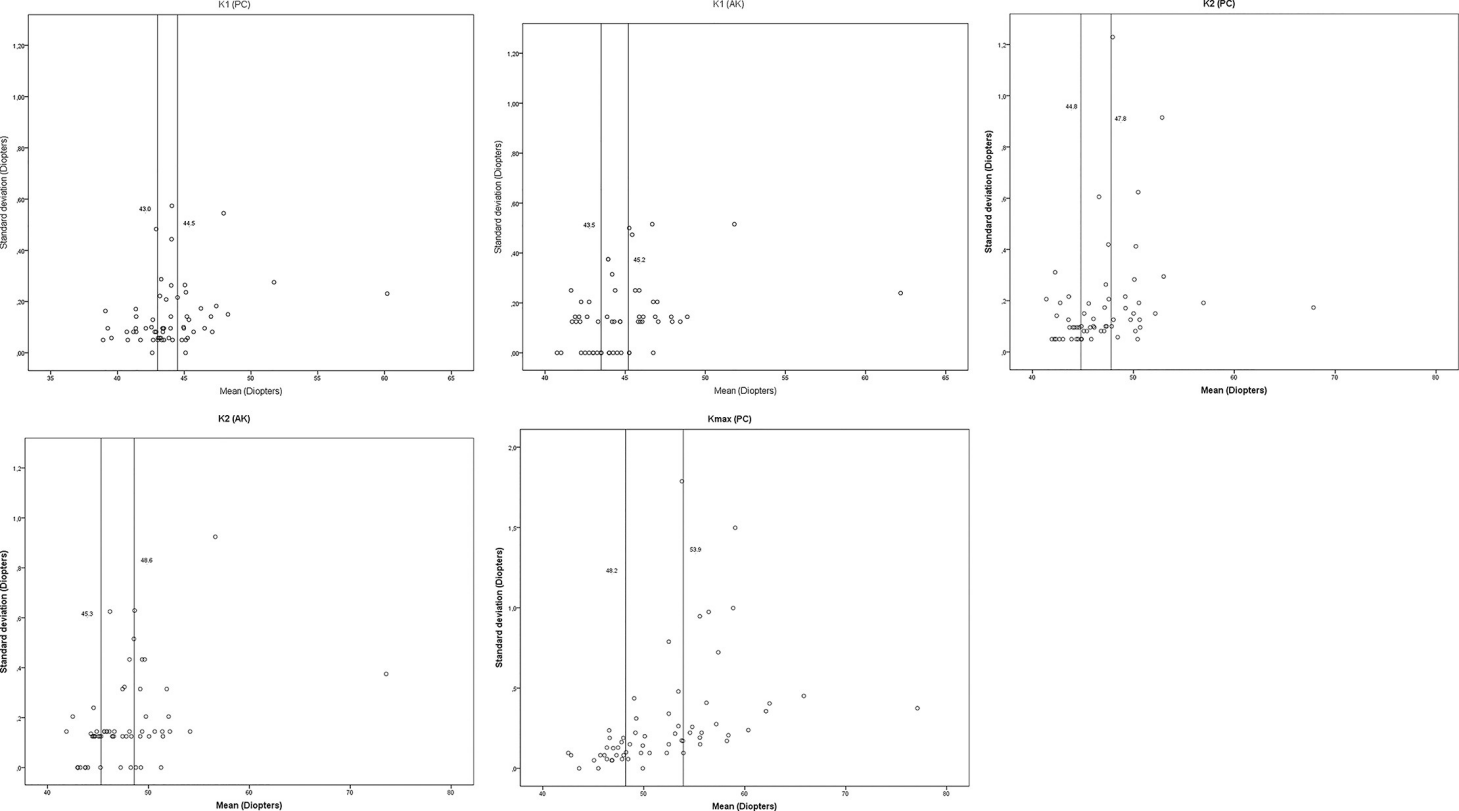
Mean values of the flattest central keratometry value (K1) obtained with the PC (a), and the AK (b), the steepest keratometry value (K2) obtained with the PC (c), and the AK (d), and the maximum keratometry value (Kmax), plotted against the standard deviation (e). The reference lines indicate the division into groups according to Table 1.

To interpret the effect of the clinical correlation between the magnitude of the keratometric parameters and their standard deviations, the repeatability coefficients were calculated. Measurements were stratified on three levels based on parameter magnitude (disease severity) and the repeatability coefficient was calculated for each group (Table 1). As can be expected from the Tau-b data, Kmax showed the greatest discrepancy in repeatability between groups, ranging from 0.32 to 1.62 D. K2 had a smaller discrepancy, ranging from 0.35 to 1.11 D using the PC, and from 0.32 to 0.95 D using the AK. K1 showed the smallest discrepancy between groups, ranging from 0.40 to 0.57 D (PC) and from 0.34 to 0.70 D (AK) (Table 1). Fig 1 shows the mean values for each parameter.

### Agreement

**Fig 2:**
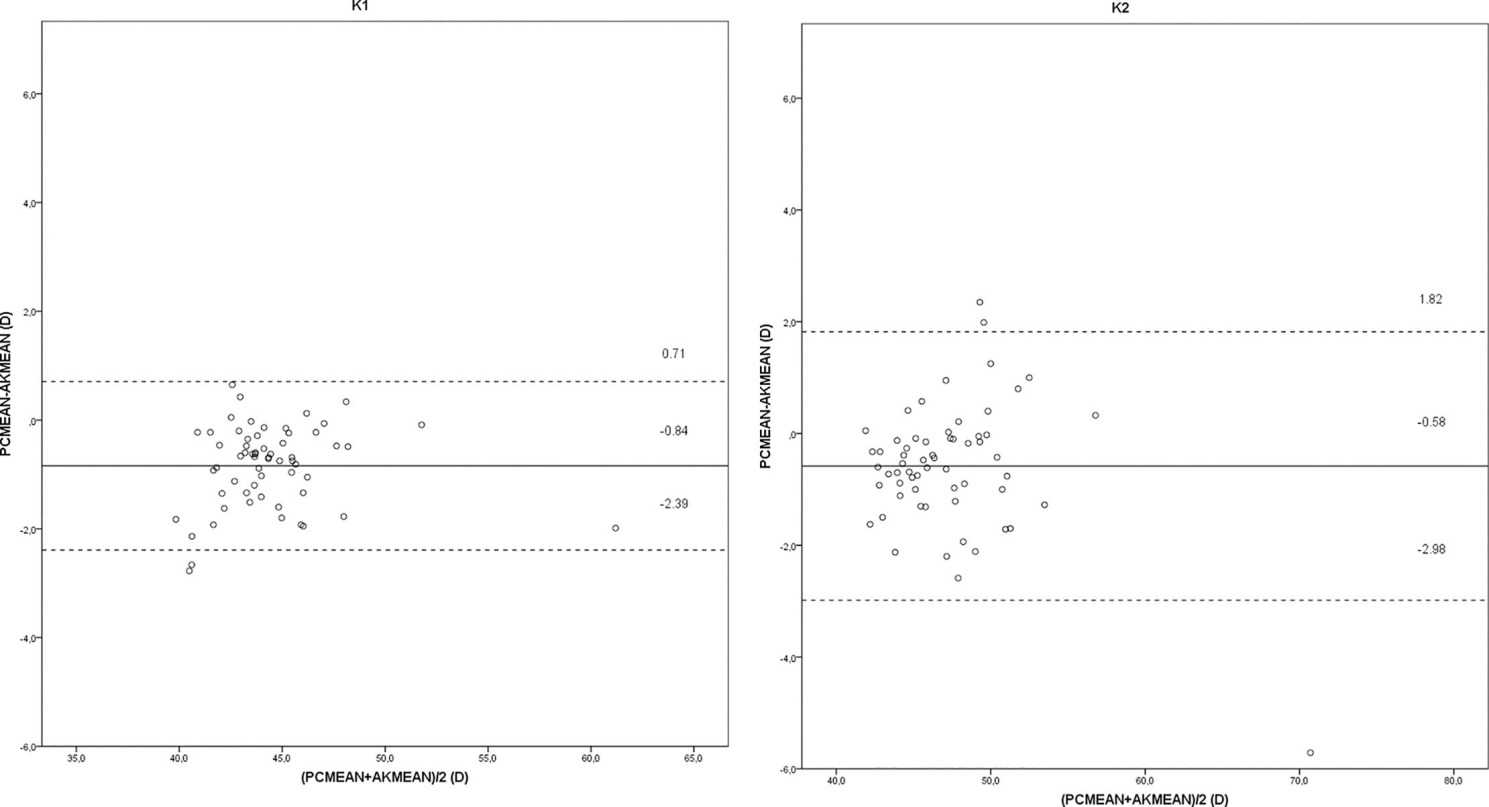
Mean difference, with 95% limits of agreement, between the two instruments, the PC (Pentacam HR) and the AK (Nidek ARK-560A) for: (a) the flattest central keratometry value (K1), and (b) the steepest central keratometry value (K2).

Fig 2 illustrates the agreement between the PC and AK instruments for K1 and K2. It can be seen that the AK system estimates higher keratometric values than the PC.

## Discussion

The findings of this study demonstrated a statistically significant association between measurement error and disease severity in terms of the magnitude of keratometric parameters in patients with keratoconus. These variations in measurement uncertainty for various degrees of severity of keratoconus should be considered when defining progression of the disease. To the best of our knowledge, this has not been attempted previously. Some previous studies have found poorer repeatability in cohorts of patients with more advanced disease. Flynn et al. [10] suggested such a relationship for Kmax, but not for K1, K2 or MCT, whereas Hashemi et al. [11] reported poorer repeatability in measurements of K1 and K2 in a cohort with K2 > 55 D (Kmax was not investigated). In these studies the focus was on describing the differences between cohorts, rather than on analysing the behaviour of the parameter per se. Kmax is probably the parameter most often used for the detection of progressive keratoconus [21], and is thus of particular interest. In the current study, a limit of 1.23 D for Kmax (95% CI 1.10–1.35 D) was found to indicate a true change between measurements. However, the effect of stratifying limits, based on disease severity, is clinically relevant. In patients with less severe disease (Kmax<48.2 D) a true change could be detected at a limit as low as 0.32 D (95% CI 0.26–0.37 D). However, the limit should be increased to 1.33 D (95% CI 1.10–1.56 D) in patients with Kmax ≥48.2 D<53.9 D, and to 1.62 D (95% CI 1.33–1.91 D) in patients with Kmax ≥53.9 D. There is thus a five-fold difference in the ability to detect a true change between consecutive measurement in the group with the least severe and the group with the most severe disease in this cohort. As a single limit is often used for all patients, usually an increase of ≥ 1 D in Kmax [21], this finding is of considerable clinical importance. Less severe disease could be misinterpreted as non-progressive, while more severe disease could be erroneously diagnosed as progressive. Patients with less severe disease, but with preserved vision, could be especially at risk of delayed referral for CXL, and thus the risk of visual deterioration. Patients with more severe disease would instead be at risk of undergoing CXL unnecessarily, and being exposed to the risk of treatment-associated side effects.

The parameters associated with central keratometry, K1 and K2, showed a higher degree of repeatability and a lower association with disease severity than did Kmax. The most repeatable parameter was K1. When measuring K1 with the PC, a difference between measurements could be detected at 0.51 D (95% CI 0.46–0.56) in the cohort as a whole, and when using the AK at 0.54 D (95% CI 0.48–0.59 D), with no significant difference between the two instruments (p = 0.130). Due to the relatively low, but significant, association between the measurement error and the parameter magnitude, stratified limits ranged from 0.40 to 0.57 D for the PC and from 0.34 to 0.70 D for the AK.

K2 had a significantly worse repeatability than K1. When measured with the PC, the limit for the cohort as a whole was 0.76 D (95% CI 0.68–0.83 D), and the stratified limits ranged from 0.35 to 1.11 D. The corresponding values obtained with the AK were 0.69 D (95% CI 0.62–0.76 D) and a range in limits from 0.32 to 0.95 D.

To the best of our knowledge, no studies have been carried out on the repeatability of AK measurements in subjects with keratoconus. K1 and K2 are measured in the central 3 mm (PC) or 3.3 mm (AK) zone of the cornea, and may thus not cover the cone area. Kmax, on the other hand, is measured over the cone area, but has poorer repeatability. It would be interesting to investigate whether central keratometry, and especially K1, could play a more important role in the detection of disease progression, and if such a commonly used instrument as the AK could be used. The findings of the present study show that measurements of K1 and K2 with the two instruments are not interchangeable, due to wide limits of agreement.

In contrast to the keratometric parameters, no statistically significant correlation was found between the magnitude of r-min and MCT and their associated measurement errors. This may be advantageous, since the error in these measurements does not depend on disease severity. However, the coefficient of variation in measurements of both r-min and MCT was poorer than that in the keratometric parameters.

One possible limitation of this study is the under-representation of females (11.5%). No studies have been published on the prevalence of keratoconus in Sweden. Previous investigations from various parts of the world, including North America [22], China [23] and Saudi Arabia [24], have found no evidence of a gender-associated prevalence of keratoconus. However, it was concluded in a Dutch study that there was a 60.6% male predominance [25], similar to 66.9%, in a recently published Danish study [26]. All diagnoses made at Swedish hospitals are recorded in patient registers. A search was carried out to identify all diagnoses of keratoconus at our university hospital between the years 2014 and 2018, showing that 1759 patients had been diagnosed with keratoconus, 454 of whom were female (25.8%). The proportion of females ranged from 21–27% over this period. This finding could indicate a lower prevalence of keratoconus in Swedish females, suggesting that further studies should be carried out on the gender distribution of keratoconus in Sweden.

In conclusion, we have demonstrated that measurement uncertainties increase with disease severity, i.e., the magnitude of keratometric parameters. Stratified repeatability limits were therefore calculated based on disease severity. Less severe disease could be misinterpreted as non-progressive, while more severe disease could be erroneously diagnosed as progressive if a single limit is used for all patients. However, it is important to emphasize that these findings require further evaluation before they can be applied in clinical practice. As progression in keratoconus is diagnosed over time, future investigations must be performed on inter-day repeatability, stratified according to the severity of keratoconus disease. This would be an important step towards understanding true progression, and reaching consensus on the definition of progressive keratoconus.

## Supporting information

S1 File SPSS spreadsheet. Data underlying the findings.
(SAV)
